# Association of Nut Consumption with Risk of Stroke and Cardiovascular Disease: The Million Veteran Program

**DOI:** 10.3390/nu13093031

**Published:** 2021-08-30

**Authors:** Kerry L. Ivey, Xuan-Mai T. Nguyen, Rachel M. Quaden, Yuk-Lam Ho, Kelly Cho, J. Michael Gaziano, Luc Djoussé

**Affiliations:** 1 Massachusetts Veterans Epidemiology and Research Information Center (MAVERIC), Boston Veterans Affairs Healthcare System, Boston, MA 02130, USA; Xuan-Mai.Nguyen@va.gov (X.-M.T.N.); Rachel.Quaden@va.gov (R.M.Q.); Yuk-Lam.Ho@va.gov (Y.-L.H.); kelly.Cho@va.gov (K.C.); michael.gaziano@va.gov (J.M.G.); Luc.Djousse@va.gov (L.D.); 2 South Australian Health and Medical Research Institute, Infection and Immunity Theme, Adelaide 5000, Australia; 3 Department of Nutrition, Harvard T.H. Chan School of Public Health, Boston, MA 02115, USA; 4 Department of Medicine, Division of Aging, Brigham and Women’s Hospital, Boston, MA 02115, USA; 5 Department of Medicine, Harvard Medical School, Boston, MA 02115, USA; 6 Department of Medicine, Division of General Internal Medicine, Brigham and Women’s Hospital, Boston, MA 02115, USA

**Keywords:** food frequency questionnaire, nuts, cardiovascular disease, stroke, coronary artery disease

## Abstract

Cardiovascular disease (CVD), including stroke and coronary artery disease (CAD), is the major cause of mortality for Americans. Nuts have been shown to improve a variety of cardiovascular disease risk factors. This study aimed to test the hypothesis that nut consumption is inversely associated with risk of incidence of stroke, CAD, and CVD mortality in the prospective Million Veterans Program (MVP). A total of 179,827 MVP participants enrolled between 2011 and 2018 were free of CVD prior to assessment of nut consumption via the food frequency questionnaire. Incident stroke and CVD events were ascertained from the Veterans Affairs electronic medical health records and the National Death Index. We used the Cox regression model to compute multivariable adjusted hazard ratios. Over the 3.5-year median follow-up, 3362 new cases of ischemic stroke were identified. When compared with participants who rarely or never consumed nuts, those consuming nuts ≥ 5 times per week were 19% less likely to experience a stroke (95% CI: 8% to 28%); 22% less likely to suffer from CAD (95% CI: 16% to 28%); and 24% less likely to die from CVD (95% CI: 7% to 37%). Consumption of peanut butter was not associated with risk of stroke. Increased dietary intake of nuts, but not peanut butter, was associated with a lower risk of stroke, CAD, and CVD death.

## 1. Introduction

Coronary artery disease (CAD) and stroke remain the leading causes of death and disability in the US [1], despite advances in biomedical research, including the discovery of effective drugs to reduce risk factors (hypertension and lipids). This underscores the importance of primary prevention for cardiovascular disease (CVD).

Diet is a modifiable factor, and previous observational and trial data have shown beneficial effects of healthy dietary patterns on CVD risk [2]. For example, the PrediMED trial has reported a reduced risk of CVD events following an intervention with a Mediterranean diet supplemented with olive oil or nuts [3]. Some, but not all, short-term trials have reported beneficial effects of nuts on CVD risk factors, including diabetes, hypertension, endothelial function, and lipids [4]. However, data on the association of nuts with CAD and stroke remain inconsistent [5,6,7,8], with some meta-analyses showing benefits only in women or with limited amounts of nut consumption [9].

Due to limited statistical power, few studies have examined the relation of nut consumption with types of stroke (hemorrhagic vs. ischemic stroke). Lastly, while some studies reported a lower risk of fatal CAD, others found no benefits for non-fatal CAD. Of note is that most reported studies had limited statistical power to detect small yet meaningful effect size of nut consumption on CAD or stroke subtypes. Taking advantage of the MVP mega data (with several thousand CAD and stroke events), this study aimed to address the above gaps in the literature with adequate statistical power in order to define and describe the relation of frequency of nut consumption with the risk of stroke in US adult veterans.

## 2. Materials and Methods

### 2.1. Study Population

Between January 2011 and November 2018, 702,740 veterans enrolled in the Million Veteran Program (MVP) [10]. Of those enrolled, 335,754 people returned an MVP Lifestyle Survey, and within this survey, 321,407 participants provided a response to the question regarding their habitual frequency of nut consumption. After excluding participants with missing data for age, gender, race, BMI, exercise frequency, smoking status, drinking status, diabetes status, hypertension status, education or Dietary Approaches to Stop Hypertension (DASH) score, there were 266,339 eligible participants. We also excluded 16,425 participants without a Veterans Health Administration (VHA) visit after the MVP Lifestyle Survey completion date. Finally, 70,087 participants with prevalent CAD or stroke at the time of survey completion were excluded, resulting in a sample size for the primary analysis of 179,827. A secondary analysis examining associations with the frequency of peanut butter included 178,772 participants following the exclusion of individuals with missing peanut butter responses. Consent was obtained in accordance with all VA policies and under the authority of the VA Central IRB [10].

### 2.2. Assessment of Nut Intake

As part of the MVP Lifestyle Survey, a food frequency questionnaire (FFQ) was administered in order to assess habitual dietary intake over the preceding year. Participants were asked the average consumption frequency of 67 different foods, including how frequently they consumed a small packet, or one ounce, of nuts as well as how frequently 1 tablespoon of peanut butter was consumed. Possible responses included: never or less than once a month; 1 to 3 per month; once a week; 2 to 4 per week; 5 to 6 per week; once (1) a day; 2 to 3 per day; 4 to 5 per day; or ≥6 per day. 

### 2.3. Outcome Ascertainment

Non-fatal event outcomes were defined by International Statistical Classification of Diseases Ninth Revision (ICD9) codes and Tenth Revision (ICD10) codes pulled from the Veterans Affairs electronic health records housed within the Corporate Data Warehouse (CDW) [11]. Fatal event outcomes were defined by ICD9 and ICD10 codes pulled from the National Death Index (NDI) [12]. The primary outcomes for this study were acute ischemic stroke (AIS) and hemorrhagic stroke (HS). AIS was defined as non-fatal AIS or fatal AIS events using ICD9 codes 433.x, 434.x, 436.x, 437.0x, 437.6x and ICD10 codes I63.x, I65.x, I66.x, I67.2x, I67.8x, I67.6x. HS was defined as non-fatal HS or fatal HS events using ICD9 codes 430.x, 431.x and ICD10 codes I60.x, I61.x. Combined stroke was defined as non-fatal and fatal ICD9 and ICD10 codes for AIS and HS. 

The secondary outcomes were coronary artery disease (CAD) and fatal cardiovascular disease (CVD). Coronary artery disease was defined as non-fatal myocardial infarction or CAD events using ICD9 codes 410.x–411.x, 413.x–414.x, and ICD10 codes I20.x–I25.x, except I25.2, within CDW. The secondary outcome was defined as either non-fatal CAD events or fatal CAD events coded as underlying cause of death using ICD10 codes I20.x–I25.x from the NDI, or coronary angioplasty and coronary revascularization using procedural codes (CPT) 33510–33536, 9292x, 9293x, 9294x, 92973, 92974 and 92975 or ICD9 Procedure codes 36.x and 00.66. The secondary outcome of fatal CVD events were defined as ICD10 codes I10.x–I13.x, I16.x, I20.x–I25.x, I27.x, I28.x, I34.x–I37.x, I42.x, I44.x–I51.x, I60.x–I63.x, I65.x–I75.x, I77.x, 178.x, I97.x, I99.x, R00.x, R58.x, G45.x from NDI.

### 2.4. Assessment of Covariates

Participants’ age, gender, ethnicity, race, education status, height, weight, smoking status, alcohol intake, and level of physical activity were taken from the MVP Baseline and Lifestyle Surveys and supplemented from the CDW when possible. Using the FFQ responses, we constructed a modified Dietary Approaches to Stop Hypertension (DASH) score that excluded nuts based on the following 7 components: fruits, vegetables, legumes, wholegrain, sweetened beverages, dairy, and red and processed meats [13]. Participants also reported the frequency of physical activity and their body weight. Body mass index (BMI) was calculated as weight (kg)**/** height (m)^2^. 

### 2.5. Statistical Analysis 

Person-time for follow-up was computed from time of FFQ completion until the first occurrence of a fatal or non-fatal stroke event, coronary procedure or censoring time (time of death for deceased subjects, last date of update of medical records in EHR or October 2018 as the closure date of current data set if the last visit took place after October 2018). Cox proportional hazard models were implemented in order to determine the hazard ratios (HRs) and 95% confidence intervals (CIs), with participants who consumed nuts less than once per month being the reference group. We conducted age-adjusted as well as multivariate-adjusted models that adjusted for: age, age*age, sex, race, body mass index, smoking status, frequency of alcohol intake, level of physical activity, level of education, and the modified DASH score. Nut consumption frequency was entered into the model as an ordinal term to assess linearity, or non-linearity, of observed associations. We repeated this analysis to include frequency of peanut butter consumption using the same methods as described for nut intake.

We then sought to identify if the association between nut intake and the likelihood of having a stroke was modified by sex, race (White vs. Black), or age (stratified as ≥65 vs. <65 years old) by using a product term between the effect modifier and nut intake. In secondary analyses, we repeated the aforementioned analyses for the secondary outcomes of coronary artery disease (CAD) and fatal CVD. 

In order to visualize the shape of association between nut intake and stroke risk, we fitted restricted cubic splines to the proportional hazards regression models to examine non-parametrically the relation of nut intake with the likelihood of having a stroke [14]. All analyses were conducted using SAS Enterprise Guide 7.1, and the level of significance was set at *p*-value < 0.05. 

## 3. Results

### 3.1. Participant Characteristics

The median nut intake of the 179,827 MVP participants included in the study was four times per month (once per week). Their mean age was 64 (SD = 12.0) years (range: 20 to >100 years), 90% were men, 85% were Caucasians, and 11% were African American (Table 1). During the median follow up of 3.5 years, 3362 new cases of ischemic stroke and 279 new cases of hemorrhagic stroke were identified. 

### 3.2. Association of Nut Intake with Risk of Stroke

**Figure 1:**
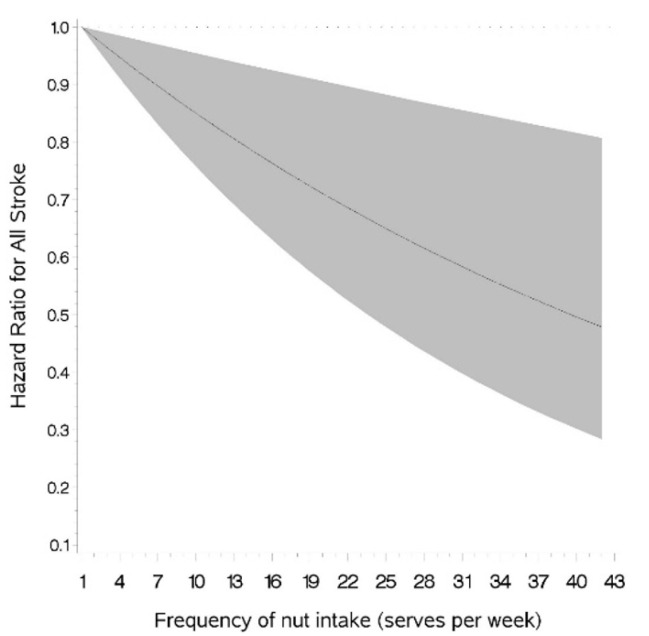
Association of nut intake and risk of total stroke.

Frequent consumption of nuts was significantly and linearly associated with a lower risk of experiencing a stroke of any type during the follow-up period in unadjusted, age-adjusted and multivariable-adjusted models (Table 2 and Figure 1). The linear association of nut intake with the risk of AIS was similar to that of total stroke (Supplementary Figure S1). Specifically, when compared to the participants who rarely/never consumed nuts, the multivariate-adjusted odds of experiencing AIS in those consuming nuts at least five times per week was 0.81 (0.72, 0.92; Table 2). The association of nut intake with the risk of HS was weaker than that for AIS. Although frequency of nut consumption was significantly and inversely related to HS risk in unadjusted and age-adjusted models, this relationship was attenuated following multivariate adjustment (Table 2).

No significant effect modification by age, sex, or race was observed for the association of nut intake with either total stroke risk, or risk of AIS or HS (data not shown).

Multivariate-adjusted model including adjustment for age, age*age, sex, race, body mass index, smoking status, frequency of alcohol intake, level of physical activity, level of education and the modified Dietary Approaches to Stop Hypertension (minus nuts) score.

### 3.3. Association of Peanut Butter Intake with Risk of Stroke

There was no evidence for an association between peanut butter consumption and risk of total stroke, AIS, or HS in any of the unadjusted or adjusted models (Table 3).

### 3.4. Association of Nut Intake with Risk of Coronary Artery Disease (CAD) and Mortality Form Cardiovascular Disease

In multivariate-adjusted Cox models, participants consuming nuts at least five times per week were 21% (95% CI: 15–27%) less likely to have experienced a CAD event and 24% (95% CI: 7–38%) less likely to have died from CVD in the follow-up period when compared with participants who never or rarely consumed nuts (Table 4).

## 4. Discussion

In this cohort of adult US veterans, we observed that in addition to being at lower risk of CAD events and CVD death, frequent nut consumers were at lower risk of experiencing a stroke. This relation was not modified by age, sex, or race and was not observed in frequent consumers of peanut butter.

Our observation that male and female adults of any race and age were at lower risk of experiencing a CHD event may be explained by the beneficial effect of nuts on CHD risk factors that have been well described in the literature [15,16,17]. Despite being a source of oxalates, which have the potential to bind to calcium and other minerals [18], nuts are rich in monounsaturated and polyunsaturated fatty acids, protein, fibers, plant sterols, vitamins, and minerals. Nuts also contain non-nutritive food components, such as L-arginine, resveratrol, phytosterols, flavonoids, and phenolic acids. This unique nutrient profile is what has been implicated in their health benefits [19,20,21,22]. Our findings suggesting that cardioprotection may be observed in frequent consumers of nuts are in line with the 2020–2025 Dietary Guidelines for Americans [23], which recommend the consumption of nuts and other nutrient dense foods as a means to meeting dietary requirements for protein and other nutrients. The benefits of nuts as a component of a healthy diet have been highlighted in a meta-analysis of randomized controlled trials [24] that found that the substitution of red meat with nuts and other high-quality plant foods such as soy and legumes resulted in improvements in CVD risk factors.

Our observation of a beneficial association of higher nut consumption on CVD could be partially attributable to the biologic effects of nuts on CVD risk factors. For example, in addition to having the potential to improve serum lipid profiles [25,26] by reducing both total and low-density lipoprotein cholesterol [20], nuts may also have the capacity to reduce apolipoprotein B concentrations in a linear dose–response fashion [27]. There is also evidence that nuts could improve vascular function [17]. One mechanism whereby nuts may improve vascular function is through the effects of L-arginine, which is a precursor of nitric oxide, a potent vasodilator responsible for regulating vascular tone and blood pressure [28]. Although data are inconsistent as to whether nuts reduce blood pressure [27,29], it is hypothesized that magnesium, potassium, and calcium may promote blood-pressure-lowering benefits [15]. Future studies contrasting effects attributable to the different varieties and types of nuts, as well as to the nutritive and non-nutritive components of nuts, are warranted in order to fully elucidate the mechanisms most relevant to reductions in CVD risk.

Despite observing an inverse association of nut intake with the risk of stroke, we did not observe a similar association for peanut butter intake. In fact, there was no association between the frequency of nut intake and the risk of total stroke or risk of stroke subtypes. These findings mimic those of a meta-analysis [30], which found that nut intake was related to lower overall and cause-specific mortality, but peanut butter was not. Although historically, this lack of association with peanut butter has been put down to the addition of partially hydrogenated vegetable fats (trans fats) to peanut butter [30,31], this may no longer be the case given the dwindling use of trans fats in the US food supply following a decision by the US Food and Drug Administration (FDA) in 2015 to revoke the “Generally Recognized As Safe” status of partially hydrogenated oils [32]. An accompanying explanation for the lack of beneficial association observed for peanut butter may be the higher sugar and saturated fatty acid content in peanut butter when compared to peanuts (not further specified) [33].

It is important to note that future studies are needed in order to validate these findings, particularly in relation to the effect size. This is because this was an observational study with measurement errors that are inherent to food frequency questionnaires in addition to unmeasured or residual confounding. Moreover, given the observational nature of this study, it is important to note that causality of observed relationships cannot be established. When considering the impact of frequent nut consumption on human health, it is important to consider the impact this may have on planetary health, as nut production [34], as well as the processes involved in delivery to consumers [35], has implications for enhanced pesticide, fuel, and fertilizer requirements, the holistic impacts of which are important to consider in relation to what dietary constituents nuts would replace.

In summary, in this prospective cohort study of male and female US veterans, we found a beneficial relationship between increased dietary intake of nuts and lower risk of stroke. However, these beneficial associations did not extend to peanut butter. More frequent intake of nuts was also associated with a lower risk of CAD and fatal CVD. Future adequately powered, large, prospective cohorts with repeated measures of nut intake are indicated.

## Figures and Tables

**Table 1:** Baseline characteristics, by frequency of nut consumption.

	Frequency of Nut Consumption				
	<1/Month	1–3/Month	1/Week	2–4/Week	≥5/Week
Number	37,075	50,238	35,863	33,500	23,151
CHARACTERISTICS					
Age (years *)*	64.6 ± 11.7	63.7 ± 12.1	63.5 ± 12.1	64.1 ± 11.9	65.2 ± 12.0
BMI (kg/m^2^*)*	29.2 ± 5.9	29.5 ± 5.5	29.4 ± 5.4	29.0 ± 5.2	28.3 ± 5.1
Male (%*)*	90.6	89.9	90.3	89.8	88.7
Race					
White (%*)*	84.1	83.7	84.8	84.8	86.5
Black (%*)*	11.8	11.7	10.7	10.9	9.3
Education					
<Highschool (%*)*	4.2	2.3	1.6	1.3	1.2
Highschool/GED (%*)*	26.9	20.2	16.8	14.8	12.6
College/AA/Bachelors *(*%*)*	59.9	64.4	65.3	65.1	62.9
Postgraduate degree *(*%*)*	9.0	13.2	16.3	18.8	23.4
Smoking					
Never (%*)*	25.2	30.1	31.9	33.5	35.1
Former (%*)*	50.1	51.6	51.7	52.5	53.7
Current (%*)*	24.6	18.3	16.4	14.0	11.2
Exercise					
<1 time /week (%*)*	51.6	44.2	37.7	33.2	29.7
1 time/week (%*)*	12.3	14.1	15.4	14.2	12.9
2–4 time/week *(*%*)*	23.1	28.7	32.9	36.4	37.0
≥ 5 times/week (%*)*	13.0	13.0	14.0	16.2	20.5
Peanut butter (Tbs/day *)*	0.2 ± 0.4	0.2 ± 0.4	0.2 ± 0.4	0.3 ± 0.4	0.4 ± 0.7
DASH score	18.9 ± 4.8	20.1 ± 4.6	21.4 ± 4.5	23.2 ± 4.4	25.1 ± 4.4
Alcohol					
Abstainer (%*)*	7.8	7.1	6.4	6.5	7.3
Former *(*%*)*	45.7	36.0	33.3	30.5	31.5
Current (%*)*	46.5	56.9	60.3	62.9	61.2

Results are mean ± SD (ANOVA) or % (chi square test), where appropriate. n = 179,827. Abbreviations: GED–general educational development; AA–associates degree; DASH score–Dietary Approaches to Stop Hypertension score; BMI–body mass index.

**Table 2:** Association of nut intake and risk of stroke.

	Frequency of Nut Intake					*p* Value
	<1/Month	1–3/Month	1/Week	2–4/Week	≥5/Week	(Linear Trend)
Number of participants	37,075	50,238	35,863	33,500	23,151	
Total stroke (n)	959	1036	647	546	382	
Crude	1.00 (ref)	0.79 (0.72, 0.86)	0.70 (0.63, 0.77)	0.64 (0.57, 0.71)	0.65 (0.57, 0.73)	<0.0001
Age-adjusted	1.00 (ref)	0.81 (0.74, 0.88)	0.72 (0.65, 0.79)	0.64 (0.57, 0.71)	0.62 (0.55, 0.70)	<0.0001
Multivariate-adjusted ^a^	1.00 (ref)	0.90 (0.83, 0.99)	0.85 (0.77, 0.95)	0.80 (0.72, 0.89)	0.81 (0.72, 0.92)	0.002
Atherosclerotic ischemic stroke (n)	904	981	602	516	359	
Crude	1.00 (ref)	0.79 (0.72, 0.87)	0.69 (0.62, 0.77)	0.64 (0.57, 0.71)	0.64 (0.57, 0.73)	<0.0001
Age-adjusted	1.00 (ref)	0.81 (0.74, 0.89)	0.71 (0.64, 0.78)	0.64 (0.57, 0.71)	0.62 (0.55, 0.70)	<0.0001
Multivariate-adjusted ^a^	1.00 (ref)	0.91 (0.83, 1.00)	0.84 (0.76, 0.93)	0.80 (0.71, 0.89)	0.81 (0.71, 0.92)	0.005
Hemorrhagic stroke (n)	75	71	57	76		
Crude	1.00 (ref)	0.69 (0.50, 0.96)	0.79 (0.56, 1.12)	0.67 (0.49, 0.93)		0.046
Age-adjusted	1.00 (ref)	0.71 (0.51, 0.98)	0.81 (0.57, 1.14)	0.67 (0.48, 0.91)		0.030
Multivariate-adjusted ^a^	1.00 (ref)	0.80 (0.58, 1.11)	0.99 (0.69, 1.41)	0.85 (0.61, 1.20)		0.160

Results are Hazard Ratio (95% CI) from Cox proportional hazard models. n = 179,827. ^a^ Multivariate-adjusted model includes age, age*age, sex, race, body mass index, smoking status, frequency of alcohol intake, level of physical activity, level of education and the modified Dietary Approaches to Stop Hypertension (minus nuts) score.

**Table 3:** Association of peanut butter intake and risk of stroke.

	Frequency of Nut Intake					*p* Value
	<1/Month	1–3/Month	1/Week	2–4/Week	≥5/Week	(Linear Trend)
Number of participants	43,897	44,716	35,729	35,035	19,395	
Total stroke (n)	949	873	681	645	384	
Crude	1.00 (ref)	0.90 (0.82, 0.99)	0.90 (0.81, 0.99)	0.86 (0.78, 0.95)	0.93 (0.82, 1.04)	0.94
Age-adjusted	1.00 (ref)	0.93 (0.85, 1.02)	0.90 (0.82, 0.99)	0.84 (0.76, 0.93)	0.89 (0.79, 1.00)	0.54
Multivariate-adjusted ^a^	1.00 (ref)	0.94 (0.85, 1.03)	0.93 (0.84, 1.03)	0.88 (0.79, 0.97)	0.94 (0.83, 1.06)	0.90
Atherosclerotic ischemic stroke (n)	896	814	641	612	363	
Crude	1.00 (ref)	0.89 (0.81, 0.98)	0.89 (0.81, 0.99)	0.87 (0.78, 0.96)	0.93 (0.82, 1.05)	0.76
Age-adjusted	1.00 (ref)	0.92 (0.84, 1.02)	0.90 (0.81, 0.99)	0.84 (0.76, 0.93)	0.89 (0.79, 1.00)	0.71
Multivariate-adjusted ^a^	1.00 (ref)	0.92 (0.84, 1.01)	0.92 (0.83, 1.02)	0.88 (0.79, 0.97)	0.94 (0.83, 1.06)	0.79
Hemorrhagic stroke (n)	70	71	56	78		
Crude	1.00 (ref)	1.00 (0.72, 1.39)	1.00 (0.70, 1.42)	0.91 (0.66, 1.26)		0.53
Age-adjusted	1.00 (ref)	1.03 (0.74, 1.44)	1.01 (0.71, 1.43)	0.88 (0.64, 1.22)		0.40
Multivariate-adjusted ^a^	1.00 (ref)	1.05 (0.76, 1.47)	1.07 (0.75, 1.53)	0.96 (0.69, 1.33)		0.56

Results are Hazard Ratio (95% CI) from Cox proportional hazard models. n = 178,772. ^a^ Multivariate-adjusted model includes age, age*age, sex, race, body mass index, smoking status, frequency of alcohol intake, level of physical activity, level of education and the modified Dietary Approaches to Stop Hypertension (minus nuts) score.

**Table 4:** Association of nut intake and incidence of cardiovascular disease mortality and coronary artery disease.

	Frequency of Nut Intake					*p* Value
	<1/Month	1–3/Month	1/Week	2–4/Week	≥5/Week	(Linear Trend)
Number of participants	37,075	50,238	35,863	33,500	23,151	
Coronary artery disease (n)	2501	2911	1877	1599	1020	
Crude	1.00 (ref)	0.85 (0.81, 0.90)	0.78 (0.73, 0.82)	0.71 (0.67, 0.76)	0.66 (0.61, 0.71)	<0.0001
Age-adjusted	1.00 (ref)	0.87 (0.82, 0.92)	0.79 (0.75, 0.84)	0.71 (0.67, 0.76)	0.64 (0.59, 0.69)	<0.0001
Multivariate-adjusted ^a^	1.00 (ref)	0.93 (0.89, 0.99)	0.89 (0.84, 0.95)	0.83 (0.78, 0.89)	0.78 (0.72, 0.84)	<0.0001
Cardiovascular disease mortality (n)	361	393	218	192	147	
Crude	1.00 (ref)	0.80 (0.69, 0.92)	0.63 (0.53, 0.74)	0.59 (0.50, 0.71)	0.66 (0.55, 0.80)	0.004
Age-adjusted	1.00 (ref)	0.86 (0.74, 0.99)	0.68 (0.57, 0.80)	0.61 (0.51, 0.73)	0.61 (0.50, 0.74)	0.0001
Multivariate-adjusted ^a^	1.00 (ref)	0.95 (0.82, 1.10)	0.79 (0.66, 0.94)	0.74 (0.62, 0.89)	0.76 (0.63, 0.93)	0.040

Results are Hazard Ratio (95% CI) from Cox proportional hazard models. n = 179,827. ^a^ Multivariate-adjusted model includes age, age*age, sex, race, body mass index, smoking status, frequency of alcohol intake, level of physical activity, level of education and the modified Dietary Approaches to Stop Hypertension (minus nuts) score.

## Data Availability

Data described in the article, code book, and analytic code will not be made available to other researchers for purposes of reproducing the results or replicating the procedure in order to comply with current VA privacy regulations pursuant to the US Department of Veterans Administration policies on compliance with the confidentiality of US veterans’ data.

## Supplementary Materials

The following are available online at https://www.mdpi.com/article/10.3390/nu13093031/s1, Supplementary Figure S1: Association of nut intake and risk of atherosclerotic ischemic stroke; File S1: Acknowledgements.
