# Supplementary material for: Association of Nut Consumption with Risk of Stroke and Cardiovascular Disease: The Million Veteran Program
Source: Nutrients. 2021 Aug 30;13(9):3031. doi: 10.3390/nu13093031 (PMC8472092; doi:10.3390/nu13093031)
Supplement: Supplementary file 1 [file nutrients-13-03031-s001.zip › nutrients-1326549-supplementary.pdf]

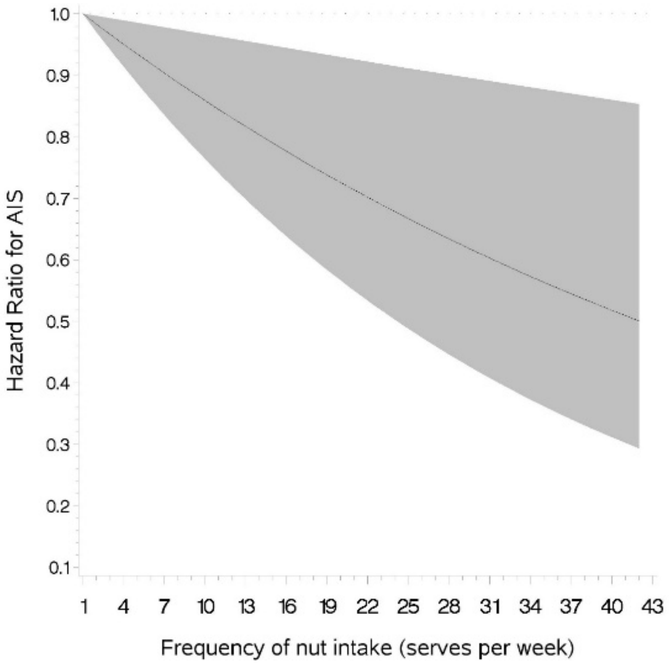

SUPPLEMENTARY INFORMATION

Supplementary Figure S1: Association of nut intake and risk of Atherosclerotic ischemic stroke

Multivariate-adjusted model including adjustment for age, age*age, sex, race, body mass index, smoking status, frequency of alcohol intake, level of physical activity, level of education and the modified Dietary Approaches to Stop Hypertension (minus nuts) score

File S1: Acknowledgements

MVP Executive Committee

- Co-Chair: J. Michael Gaziano, M.D., M.P.H.
  - VA Boston Healthcare System, 150 S. Huntington Avenue, Boston, MA 02130, USA
- Co-Chair: Sumitra Muralidhar, Ph.D.
  - US Department of Veterans Affairs, 810 Vermont Avenue NW, Washington, DC 20420, USA
- Rachel Ramoni, D.M.D., Sc.D., Chief VA Research and Development Officer
  - US Department of Veterans Affairs, 810 Vermont Avenue NW, Washington, DC 20420, USA
- Jean Beckham, Ph.D.
  - Durham VA Medical Center, 508 Fulton Street, Durham, NC 27705, USA
- Kyong-Mi Chang, M.D.
  - Philadelphia VA Medical Center, 3900 Woodland Avenue, Philadelphia, PA 19104, USA
- Christopher J. O'Donnell, M.D., M.P.H.
  - VA Boston Healthcare System, 150 S. Huntington Avenue, Boston, MA 02130, USA
- Philip S. Tsao, Ph.D.
  - VA Palo Alto Health Care System, 3801 Miranda Avenue, Palo Alto, CA 94304, USA
- James Breeling, M.D., Ex-Officio
  - US Department of Veterans Affairs, 810 Vermont Avenue NW, Washington, DC 20420, USA
- Grant Huang, Ph.D., Ex-Officio
  - US Department of Veterans Affairs, 810 Vermont Avenue NW, Washington, DC 20420, USA
- Juan P. Casas, M.D., Ph.D., Ex-Officio
  - VA Boston Healthcare System, 150 S. Huntington Avenue, Boston, MA 02130, USA

MVP Program Office

- Sumitra Muralidhar, Ph.D.
  - o US Department of Veterans Affairs, 810 Vermont Avenue NW, Washington, DC 20420, USA
- Jennifer Moser, Ph.D.
  - o US Department of Veterans Affairs, 810 Vermont Avenue NW, Washington, DC 20420, USA

MVP Recruitment/Enrollment

- Recruitment/Enrollment Director/Deputy Director, Boston—Stacey B. Whitbourne, Ph.D.; Jessica V. Brewer, M.P.H.
  - VA Boston Healthcare System, 150 S. Huntington Avenue, Boston, MA 02130, USA
- MVP Coordinating Centers
  - Clinical Epidemiology Research Center (CERC), West Haven—Mihaela Aslan, Ph.D.
  - West Haven VA Medical Center, 950 Campbell Avenue, West Haven, CT 06516, USA
  - Cooperative Studies Program Clinical Research Pharmacy Coordinating Center, Albuquerque—Todd Connor, Pharm.D.; Dean P. Argyres, B.S., M.S.
  - New Mexico VA Health Care System, 1501 San Pedro Drive SE, Albuquerque, NM 87108, USA
  - Genomics Coordinating Center, Palo Alto—Philip S. Tsao, Ph.D.
  - VA Palo Alto Health Care System, 3801 Miranda Avenue, Palo Alto, CA 94304, USA
  - MVP Boston Coordinating Center, Boston—J. Michael Gaziano, M.D., M.P.H.
  - VA Boston Healthcare System, 150 S. Huntington Avenue, Boston, MA 02130, USA
  - MVP Information Center, Canandaigua—Brady Stephens, M.S.

- Canandaigua VA Medical Center, 400 Fort Hill Avenue, Canandaigua, NY 14424, USA
- VA Central Biorepository, Boston—Mary T. Brophy M.D., M.P.H.; Donald E. Humphries, Ph.D.; Luis E. Selva, Ph.D.
- VA Boston Healthcare System, 150 S. Huntington Avenue, Boston, MA 02130, USA
- MVP Informatics, Boston—Nhan Do, M.D.; Shahpoor (Alex) Shayan, M.S.
- VA Boston Healthcare System, 150 S. Huntington Avenue, Boston, MA 02130, USA
- MVP Data Operations/Analytics, Boston—Kelly Cho, M.P.H., Ph.D.
- VA Boston Healthcare System, 150 S. Huntington Avenue, Boston, MA 02130, USA
- Director of Regulatory Affairs—Lori Churby, B.S.
- VA Palo Alto Health Care System, 3801 Miranda Avenue, Palo Alto, CA 94304, USA

MVP Science

- Science Operations—Christopher J. O'Donnell, M.D., M.P.H.
- VA Boston Healthcare System, 150 S. Huntington Avenue, Boston, MA 02130, USA
- Genomics Core—Christopher J. O'Donnell, M.D., M.P.H.; Saiju Pyarajan Ph.D.
- VA Boston Healthcare System, 150 S. Huntington Avenue, Boston, MA 02130, USA
- Philip S. Tsao, Ph.D.
- VA Palo Alto Health Care System, 3801 Miranda Avenue, Palo Alto, CA 94304, USA
- Data Core—Kelly Cho, M.P.H, Ph.D.
- VA Boston Healthcare System, 150 S. Huntington Avenue, Boston, MA 02130, USA
- VA Informatics and Computing Infrastructure (VINCI)—Scott L. DuVall, Ph.D.
- VA Salt Lake City Health Care System, 500 Foothill Drive, Salt Lake City, UT 84148, USA
- Data and Computational Sciences—Saiju Pyarajan, Ph.D.
- VA Boston Healthcare System, 150 S. Huntington Avenue, Boston, MA 02130, USA
- Statistical Genetics—Elizabeth Hauser, Ph.D.
- Durham VA Medical Center, 508 Fulton Street, Durham, NC 27705, USA
- Yan Sun, Ph.D.
- Atlanta VA Medical Center, 1670 Clairmont Road, Decatur, GA 30033, USA
- Hongyu Zhao, Ph.D.
- West Haven VA Medical Center, 950 Campbell Avenue, West Haven, CT 06516, USA

Current MVP Local Site Investigators

- Atlanta VA Medical Center (Peter Wilson, M.D.)
  - 1670 Clairmont Road, Decatur, GA 30033, USA
- Bay Pines VA Healthcare System (Rachel McArdle, Ph.D.)
  - 10,000 Bay Pines Blvd Bay Pines, FL 33744, USA
- Birmingham VA Medical Center (Louis Dellitalia, M.D.)
  - 700 S. 19th Street, Birmingham, AL 35233, USA
- Central Western Massachusetts Healthcare System (Kristin Mattocks, Ph.D., M.P.H.)
  - 421 North Main Street, Leeds, MA 01053, USA
- Cincinnati VA Medical Center (John Harley, M.D., Ph.D.)
  - 3200 Vine Street, Cincinnati, OH 45220, USA
- Clement J. Zablocki VA Medical Center (Jeffrey Whittle, M.D., M.P.H.)

- 5000 West National Avenue, Milwaukee, WI 53295, USA

- VA Northeast Ohio Healthcare System (Frank Jacono, M.D.)
  - 10701 East Boulevard, Cleveland, OH 44106, USA
- Durham VA Medical Center (Jean Beckham, Ph.D.)
  - 508 Fulton Street, Durham, NC 27705, USA
- Edith Nourse Rogers Memorial Veterans Hospital (John Wells., Ph.D.)
  - 200 Springs Road, Bedford, MA 01730, USA
- Edward Hines, Jr. VA Medical Center (Salvador Gutierrez, M.D.)
  - 5000 South 5th Avenue, Hines, IL 60141, USA
- Veterans Health Care System of the Ozarks (Gretchen Gibson, D.D.S., M.P.H.)
  - 1100 North College Avenue, Fayetteville, AR 72703, USA
- Fargo VA Health Care System (Kimberly Hammer, Ph.D.)
  - 2101 N. Elm, Fargo, ND 58102, USA
- VA Health Care Upstate New York (Laurence Kaminsky, Ph.D.)
  - 113 Holland Avenue, Albany, NY 12208, USA
- New Mexico VA Health Care System (Gerardo Villareal, M.D.)
  - 1501 San Pedro Drive, S.E. Albuquerque, NM 87108, USA
- VA Boston Healthcare System (Scott Kinlay, M.B.B.S., Ph.D.)
  - 150 S. Huntington Avenue, Boston, MA 02130, USA
- VA Western New York Healthcare System (Junzhe Xu, M.D.)
  - 3495 Bailey Avenue, Buffalo, NY 14215-1199, USA
- Ralph H. Johnson VA Medical Center (Mark Hamner, M.D.)
  - 109 Bee Street, Mental Health Research, Charleston, SC 29401
- Columbia VA Health Care System (Roy Mathew, M.D.)
  - 6439 Garners Ferry Road, Columbia, SC 29209, USA
- VA North Texas Health Care System (Sujata Bhushan, M.D.)
  - 4500 S. Lancaster Road, Dallas, TX 75216, USA
- Hampton VA Medical Center (Pran Iruvanti, D.O., Ph.D.)
  - 100 Emancipation Drive, Hampton, VA 23667, USA
- Richmond VA Medical Center (Michael Godschalk, M.D.)
  - 1201 Broad Rock Blvd., Richmond, VA 23249, USA
- Iowa City VA Health Care System (Zuhair Ballas, M.D.)
  - 601 Highway 6 West, Iowa City, IA 52246-2208
- Eastern Oklahoma VA Health Care System (Douglas Ivins, M.D.)
  - 1011 Honor Heights Drive, Muskogee, OK 74401, USA
- James A. Haley Veterans' Hospital (Stephen Mastorides, M.D.)
  - 13000 Bruce B. Downs Blvd, Tampa, FL 33612, USA
- James H. Quillen VA Medical Center (Jonathan Moorman, M.D., Ph.D.)
  - Corner of Lamont & Veterans Way, Mountain Home, TN 37684, USA
- John D. Dingell VA Medical Center (Saib Gappy, M.D.)
  - 4646 John R Street, Detroit, MI 48201, USA

- Louisville VA Medical Center (Jon Klein, M.D., Ph.D.)
  - 800 Zorn Avenue, Louisville, KY 40206, USA
- Manchester VA Medical Center (Nora Ratcliffe, M.D.)
  - 718 Smyth Road, Manchester, NH 03104, USA
- Miami VA Health Care System (Hermes Florez, M.D., Ph.D.)
  - 1201 NW 16th Street, 11 GRC, Miami, FL 33125, USA
- Michael E. DeBakey VA Medical Center (Olaoluwa Okusaga, M.D.)
  - 2002 Holcombe Blvd, Houston, TX 77030, USA
- Minneapolis VA Health Care System (Maureen Murdoch, M.D., M.P.H.)
  - One Veterans Drive, Minneapolis, MN 55417, USA
- N. FL/S. GA Veterans Health System (Peruvemba Sriram, M.D.)
  - 1601 SW Archer Road, Gainesville, FL 32608, USA
- Northport VA Medical Center (Shing Shing Yeh, Ph.D., M.D.)
  - 79 Middleville Road, Northport, NY 11768, USA
- Overton Brooks VA Medical Center (Neeraj Tandon, M.D.)
  - 510 East Stoner Ave, Shreveport, LA 71101, USA
- Philadelphia VA Medical Center (Darshana Jhala, M.D.)
  - 3900 Woodland Avenue, Philadelphia, PA 19104, USA
- Phoenix VA Health Care System (Samuel Aguayo, M.D.)
  - 650 E. Indian School Road, Phoenix, AZ 85012, USA
- Portland VA Medical Center (David Cohen, M.D.)
  - 3710 SW U.S. Veterans Hospital Road, Portland, OR 97239
- Providence VA Medical Center (Satish Sharma, M.D.)
  - 830 Chalkstone Avenue, Providence, RI 02908, USA
- Richard Roudebush VA Medical Center (Suthat Liangpunsakul, M.D., M.P.H.)
  - 1481 West 10th Street, Indianapolis, IN 46202, USA
- Salem VA Medical Center (Kris Ann Oursler, M.D.)
  - 1970 Roanoke Blvd, Salem, VA 24153, USA
- San Francisco VA Health Care System (Mary Whooley, M.D.)
  - 4150 Clement Street, San Francisco, CA 94121, USA
- South Texas Veterans Health Care System (Sunil Ahuja, M.D.)
  - 7400 Merton Minter Boulevard, San Antonio, TX 78229, USA
- Southeast Louisiana Veterans Health Care System (Joseph Constans, Ph.D.)
  - 2400 Canal Street, New Orleans, LA 70119, USA
- Southern Arizona VA Health Care System (Paul Meyer, M.D., Ph.D.)
  - 3601 S 6th Avenue, Tucson, AZ 85723, USA
- Sioux Falls VA Health Care System (Jennifer Greco, M.D.)
  - 2501 W 22nd Street, Sioux Falls, SD 57105, USA
- St. Louis VA Health Care System (Michael Rauchman, M.D.)
  - 915 North Grand Blvd, St. Louis, MO 63106, USA
- Syracuse VA Medical Center (Richard Servatius, Ph.D.)

- 800 Irving Avenue, Syracuse, NY 13210, USA

- VA Eastern Kansas Health Care System (Melinda Gaddy, Ph.D.)
    - 4101 S 4th Street Trafficway, Leavenworth, KS 66048, USA
- VA Greater Los Angeles Health Care System (Agnes Wallbom, M.D., M.S.)
    - 11301 Wilshire Blvd, Los Angeles, CA 90073, USA
- VA Long Beach Healthcare System (Timothy Morgan, M.D.)
    - 5901 East 7th Street Long Beach, CA 90822, USA
- VA Maine Healthcare System (Todd Stapley, D.O.)
    - 1 VA Center, Augusta, ME 04330, USA
- VA New York Harbor Healthcare System (Scott Sherman, M.D., M.P.H.)
    - 423 East 23rd Street, New York, NY 10010, USA
- VA Pacific Islands Health Care System (George Ross, M.D.)
    - 459 Patterson Rd, Honolulu, HI 96819, USA
- VA Palo Alto Health Care System (Philip Tsao, Ph.D.)
    - 3801 Miranda Avenue, Palo Alto, CA 94304-1290, USA
- VA Pittsburgh Health Care System (Patrick Strollo, Jr., M.D.)
    - University Drive, Pittsburgh, PA 15240, USA
- VA Puget Sound Health Care System (Edward Boyko, M.D.)
    - 1660 S. Columbian Way, Seattle, WA 98108-1597, USA
- VA Salt Lake City Health Care System (Laurence Meyer, M.D., Ph.D.)
    - 500 Foothill Drive, Salt Lake City, UT 84148, USA
- VA San Diego Healthcare System (Samir Gupta, M.D., M.S.C.S.)
    - 3350 La Jolla Village Drive, San Diego, CA 92161, USA
- VA Sierra Nevada Health Care System (Mostaqul Huq, Pharm.D., Ph.D.)
    - 975 Kirman Avenue, Reno, NV 89502, USA
- VA Southern Nevada Healthcare System (Joseph Fayad, M.D.)
    - 6900 North Pecos Road, North Las Vegas, NV 89086, USA
- VA Tennessee Valley Healthcare System (Adriana Hung, M.D., M.P.H.)
    - 1310 24th Avenue, South Nashville, TN 37212, USA
- Washington DC VA Medical Center (Jack Lichy, M.D., Ph.D.)
    - 50 Irving St, Washington, D. C. 20422, USA
- W.G. (Bill) Hefner VA Medical Center (Robin Hurley, M.D.)
    - 1601 Brenner Ave, Salisbury, NC 28144, USA
- White River Junction VA Medical Center (Brooks Robey, M.D.)
    - 163 Veterans Drive, White River Junction, VT 05009, USA
- William S. Middleton Memorial Veterans Hospital (Robert Striker, M.D., Ph.D.)
    - 2500 Overlook Terrace, Madison, WI 53705, USA
